# Bann and Aksoy Respond to “Religious Service Attendance and Public Health”

**DOI:** 10.1093/aje/kwab135

**Published:** 2021-05-11

**Authors:** David Bann, Ozan Aksoy

Estimating the causal effect of religiosity on health is challenging. Even for service attendance, for which evidence is strongest as VanderWeele et al (1) elegantly summarize, the “exposure” itself and likely its effects are enormously varied across and within religious traditions. An effect of a given type of attendance may also differ in direction and magnitude by context—cross-sectional analysis notwithstanding, associations seemingly range from negative in China to particularly positive in the United States (2). Prior findings (1, 3) that do suggest, in a given context, beneficial effects on certain health outcomes should also be interpreted with caution: What may have been the case in the past does not necessarily determine what could or indeed should be the case in the future. The somewhat acerbic reactions to past work on this topic (4–6) perhaps highlight the polarizing views on the net benefit to individuals and society that religious institutions bring (7, 8). A robust link between service attendance and health could, however, have implications for both religious and secular societies. This broader utilitarian ground may bridge opposing views and be most fruitful in terms of etiology and translation. Building on prior discussion (9), we pose 2 sets of questions that could aid such understanding.

Assuming a causal link exists, why is religious service attendance beneficial for health? What elements of attendance are most beneficial? How can those elements be transferred to other domains? Addressing these questions will not only improve the causal credibility of earlier findings but also help to improve the transferability of inferences to intervention or policy. It would also help to incorporate such “exposures” in broader environments than the US-focused context discussed by VanderWeele et al. For instance, where religious attendance may attract ostracization or hostility (10), population wellbeing could be similarly improved if nonreligious community participation is facilitated. Such alternatives will also be important given the increasing secularization observed nearly globally (11, 12). Investigating emerging secular alternatives (e.g., the Sunday Assembly) and contrasting them with religious attendance could be a first step to addressing the above questions, though such initiatives are still in their infancy (3). New data collections on secular alternatives may be warranted alongside analysis of prominent nonreligious community participation activities that are more tractably investigated in existing epidemiologic studies (e.g., sports teams and volunteer groups).

Assuming a robust set of exposures is extracted from religious service attendance, the second set of questions concerns the identification of policy levers through which these exposures could be implemented and evaluated in an inclusive way. VanderWeele et al. (1) focused on clinician-patient interactions; yet, despite their apparent neutral character, the proposed questions on religion/spirituality might be interpreted as inappropriate or even proselytization in routine care, given the clinician-patient power dynamic. A more inclusive approach could be to focus on community participation, inclusive of religious service attendance. This could be empirically tested as a form of social prescribing. More broadly, interventions focusing on such clinical interactions are likely less suitable to contexts with limited patient-clinician interactions or when such interactions necessarily focus on active treatment rather than prevention. There may be other possible upstream public health targets to improve community participation that are more effective and equitable than those operating at the individual level via activity promotion (13, 14). Epidemiologic findings thus motivate a need to better understand the determinants of and possible policy tools for facilitating community participation. Although US society has become increasingly individualized (from 1960 to 2020), a contrasting trend towards increased community involvement occurred previously (from 1900 to 1960) (15). Such shifts illustrate the potential modifiability of community participation and the possibility of incorporating the beneficial components of religious attendance in broader (religious and secular) society.
